# P52 Clinical, microbiological and outcome profile of *Enterococcus faecium* bloodstream infections in a tertiary care trauma centre in India

**DOI:** 10.1093/jacamr/dlaf230.059

**Published:** 2025-12-04

**Authors:** Parul Singh, M Nizam Ahmed, Ashish Kumar Shrivastav, Arpan Kumar Thakur, Rasna Parveen, Bharat Chandra Das, Vanlal Tluanpuii, Madhavi Kirti, Subodh Kumar, Sushma Sagar, Kapil Dev Soni, Richa Aggarwal, Ashish Bindra, Keshav Goyal, Gyanendra Pal Singh, Sokhal Navdeep, Kamran Farooque, Purva Mathur

**Affiliations:** All India Institute of Medical Sciences, New Delhi, India; All India Institute of Medical Sciences, New Delhi, India; Trauma Centre, All India Institute of Medical Sciences, New Delhi, India; Trauma Centre, All India Institute of Medical Sciences, New Delhi, India; Trauma Centre, All India Institute of Medical Sciences, New Delhi, India; All India Institute of Medical Sciences, New Delhi, India; All India Institute of Medical Sciences, New Delhi, India; All India Institute of Medical Sciences, New Delhi, India; All India Institute of Medical Sciences, New Delhi, India; All India Institute of Medical Sciences, New Delhi, India; Trauma Centre, All India Institute of Medical Sciences, New Delhi, India; Trauma Centre, All India Institute of Medical Sciences, New Delhi, India; Trauma Centre, All India Institute of Medical Sciences, New Delhi, India; Trauma Centre, All India Institute of Medical Sciences, New Delhi, India; Trauma Centre, All India Institute of Medical Sciences, New Delhi, India; Trauma Centre, All India Institute of Medical Sciences, New Delhi, India; Trauma Centre, All India Institute of Medical Sciences, New Delhi, India; All India Institute of Medical Sciences, New Delhi, India

## Abstract

**Background:**

*Enterococcus faecium* drives healthcare-associated bloodstream infections (BSIs) globally, with multidrug resistance and high mortality posing challenges, particularly in trauma settings. Scarce data from Indian trauma centres necessitate analysis to guide management.

**Objectives:**

To assess the epidemiology, clinical characteristics, antimicrobial resistance and outcomes of *E. faecium* BSIs in a tertiary trauma care facility in India.

**Methods:**

A retrospective study analysed all *E. faecium* BSI cases in ICUs of a public-sector tertiary trauma centre. Demographic, clinical, microbiological and antimicrobial susceptibility data were collected. BSIs were classified as primary (central line-associated bloodstream infections [CLABSI] or non-CLABSI) or secondary per CDC definitions. Outcomes were evaluated at 14 days and final follow-up using R software.

**Results:**

Of 97 BSI events involving 112 *E. faecium* isolates, 51.5% occurred in the trauma ICU. Median patient age was 40 years (IQR: 27–55), with 75.3% male. Median time to infection was 8 days (IQR: 5–12) post-admission. CLABSIs, primarily associated with non-tunnelled jugular lines (81.4%), accounted for most cases, followed by non-CLABSI (12.4%) and secondary BSIs (6.2%). Median ICU stay was 21 days (IQR: 14–30). Antimicrobial resistance showed 92.5% of isolates resistant to ampicillin and 37.6% non-susceptible to vancomycin. Linezolid susceptibility was 86.8% and tigecycline susceptibility was 100%. Mortality was 30.9% at 14 days and 48.4% at final follow-up. Early catheter removal occurred in 45.3% of CLABSI cases.

**Discussion:**

The predominance of CLABSIs (81.4%) aligns with global data, where *E. faecium* is a leading ICU pathogen, driven by catheter use, as seen in European and North American studies. High vancomycin resistance (37.6%) mirrors Indian (up to 81% in high-risk settings) and global (34–81%) VREfm trends, limiting therapeutic options. Linezolid and tigecycline’s efficacy is consistent with international meta-analyses but risks emerging resistance with overuse. Prolonged LOS (21 days) and high mortality (48.4%) reflect VREfm severity, comparable to Chinese (24%) and Italian (27.5–36.6%) studies. Early catheter removal, effective in North American studies, occurred in only 45.3% of CLABSI cases, suggesting underutilization in resource-constrained settings. These findings underscore the need for enhanced catheter care protocols and antimicrobial stewardship to curb MDR *E. faecium*. Future research should explore molecular resistance mechanisms and cost-effective interventions tailored to Indian trauma settings.

**Conclusions:**

*E. faecium* BSIs in Indian trauma ICUs require urgent infection control and stewardship to address high resistance and mortality.

